# Retrospective analysis of nystagmus characteristics and clinical applications of positional testing in patients with cupulolithiasis of the posterior semicircular canal in benign paroxysmal positional vertigo

**DOI:** 10.3389/fneur.2024.1413929

**Published:** 2024-07-10

**Authors:** Jing Wu, Yihuai Zou, Wenyan Xu, Hongming Ma, Lixian Huang, Bo Zhao, Liman Sun

**Affiliations:** Dongzhimen Hospital, Beijing University of Chinese Medicine, Beijing, China

**Keywords:** posterior semicircular canal cupulolithiasis, benign paroxysmal positional vertigo, positional testing, Half-Hallpike maneuver, Vertigo

## Abstract

**Objective:**

This study aimed to investigate the characteristics of positional nystagmus in patients with cupulolithiasis of the posterior semicircular canal-benign paroxysmal positional vertigo (PC-BPPV-cu) to improve clinical diagnostic accuracy.

**Methods:**

This study retrospectively analyzed 128 cases of PC-BPPV-cu and 128 cases of canalolithiasis of BPPV (PC-BPPV-ca). General data, intensity, distribution, and the correlation of positional nystagmus were compared between the two groups.

**Results:**

Compared to the PC-BPPV-ca group, more cases from the PC-BPPV-cu group initially presented in the emergency department (*P* < 0.05). The most frequent positional nystagmus induced by PC-BPPV-cu was torsional-upbeat nystagmus, characterized by the upper pole of the affected eye beating toward the lower ear and vertically upward (387 cases, 59.7%). It was followed by torsional-downbeat nystagmus, characterized by the upper pole of the unaffected eye beating toward the lower ear and vertically downward (164 cases, 25.3%). The former represented posterior canal excitatory nystagmus (PC-EN), while the latter represented posterior canal inhibitory nystagmus (PC-IN). In the PC-BPPV-cu group, PC-EN was most easily caused by the Half Dix–Hallpike (HH) maneuver on the affected side, while PC-IN was most easily induced by a face-down position (FDP) on the unaffected side at approximately 45° angle (45° FDP). The vertical slow phase velocity (v-SPV) of positional nystagmus was more potent in the affected HH than in other positions with PC-EN (all *P* < 0.05); the v-SPV of positional nystagmus was greater in the 45° FDP than in different positions with PC-IN (all *P* < 0.05); the v-SPV of the affected Dix–Hallpike (DH) maneuver in the PC-BPPV-ca group was significantly greater than that of the affected HH maneuver in the PC-BPPV-cu group (*P* < 0.05). The *a priori* analysis showed that the strongest correlation with HH positional nystagmus was observed in the affected side roll test, followed by the DH maneuver.

**Conclusion:**

In the PC-BPPV-cu group, the HH maneuver most easily induced PC-EN on the affected side, and PC-IN was most easily induced by the 45° FDP. In some cases of PC-BPPV-cu, significant nystagmus was not observed to be induced in the DH position on the affected side; however, vertical rotation nystagmus was induced in the roll-test position on the affected side. In such cases, PC-BPPV-cu diagnosis should be considered, and HH and 45° FDP tests should be conducted to support the diagnosis.

## Introduction

Cupulolithiasis of posterior semicircular benign paroxysmal positional vertigo (PC-BPPV-cu) is a rare form of benign paroxysmal positional vertigo (BPPV). Epley first proposed that the characteristic nystagmus of PC-BPPV-cu is induced in the Half Dix–Hallpike (HH) maneuver position, characterized by continuous upward and torsional nystagmus (1). As the cupula of the posterior semicircular canal (PC) may be oriented along the earth-horizontal axis, the weighted cupula could have a maximal propensity to be deflected earthward (1). PC-BPPV-cu was described in the 2015 diagnostic criteria for BPPV by the Barany Society (2). Manipulative reduction techniques commonly used for BPPV are ineffective for PC-BPPV-cu, which highlights the importance of distinguishing between cupulolithiasis and canalolithiasis classifications of PC-BPPV (3).

Moreover, some patients report persistent vertigo due to the persistent nystagmus characteristic of cupulolithiasis, which contrasts with the transient positional vertigo typical of BPPV. Consequently, PC-BPPV-cu is frequently mistaken for acute vestibular syndrome in clinical practice, necessitating early identification. This study retrospectively analyzed data collected from patients with PC-BPPV-cu and those with PC-BPPV-ca at the researchers' hospital, Tongzhou Branch of Dongzmen Hospital, over the past 4 years. The study aimed to explore the characteristics of nystagmus in PC-BPPV-cu.

## Materials and methods

### General information

Cases of patients with vertigo admitted to the Department of Encephalopathy, Tongzhou Branch of Dongzmen Hospital, Beijing, from January 2019 to December 2022 were reviewed. All PC-BPPV-cu cases that met the inclusion criteria were included. An equal number of PC-BPPV-ca cases were selected as controls using random sampling.

### Diagnosis

#### PC-BPPV-ca

The diagnostic criteria (2) for PC-BPPV-ca included the following:

A. recurrent attacks of positional vertigo or positional dizziness provoked by lying down or turning over in the supine position (SP);

B. duration of attacks being <1 min;

C. positional nystagmus elicited after a latency of 1 or a few seconds by the Dix–Hallpike maneuver (DH position) or side-lying maneuver (Semont diagnostic maneuver). The nystagmus is a combination of torsional nystagmus with the upper pole of the eyes beating toward the lower ear and vertical nystagmus beating upward (toward the forehead), typically lasting <1 min; and

D. not attributable to another disorder.

#### PC-BPPV-cu

The diagnostic criteria (2) for PC-BPPV-ca included the following:

A. recurrent attacks of positional vertigo or dizziness provoked by lying down or turning over in the SP;

B. positional nystagmus elicited after a brief or no latency by HH, beating torsionally from the upper pole of the eye to the lower ear and vertically upward (toward the forehead) and lasting >1 min; and

C. not attributable to another disorder.

#### Inclusion criteria

The inclusion criteria were as follows: (1) Age and gender were not restricted; (2) the final diagnosis was PC-BPPV-ca and PC-BPPC-cu, according to the 2015 diagnostic criteria for BPPV; (3) roll and head-hanging tests were completed in all cases to exclude BPPV in the horizontal and anterior semicircular canals; and (4) brain imaging (MRI/CT) and neurological examination did not indicate central lesions.

#### Exclusion criteria

The exclusion criteria were as follows: (1) Intracranial space-occupying lesions, postoperative craniotomy, a history of posterior circulation stroke or secondary BPPV and (2) a 48-h history of alcohol intake.

### Observation indicators

General information included age, gender, course of the disease, the first-visit department, and the affected side. Positional nystagmus data of all the cases were recorded for the following positions: DH, roll test, supine, and sitting nose-down. For PC-BPPV-cu, additional positions included HH and 45° FDP. The order of positional tests for all patients was as follows: right DH, left DH, SP, right roll test, left roll test, right HH, left HH, 45° FDP, and sitting nose-down positions. A goniometer helped us to determine the angle of the patient's head position during the positional testing.

HH (4): The maneuver is performed with turning the head of the patient to an angle of 45° toward the side to be tested, positioning it approximately flat on the examination table.

FDP on the unaffected side (45° FDP): The maneuver is performed with the patient lying on the healthy side and their head turned to an angle of 45° to face the ground. This position is located 180° opposite to the HH orientation.

DH: The patient is asked to sit upright on the examination table with their legs extended. The clinician rotates the head 45° toward the ear to be tested. The patient is then asked to lay back swiftly with the head hanging off the edge of the examination table. The patient's neck is extended to ~20° below the horizontal plane, while the head is maintained at the initial 45° rotation.

Roll test: The head is rotated approximately 90° to each side while the patient is in the SP.

Sitting nose-down position: The patient is seated, turned 45° to the healthy side, and bent forward by approximately 90°.

SP: The patient lies in the SP with the head raised by approximately 30°.

The mean of binocular vertical slow phase velocity (v-SPV) was used as an index to evaluate the intensity of nystagmus. Due to the excitatory potential of the unilateral PC, traction eye movements were caused by the ipsilateral superior oblique and contralateral inferior rectus muscles. Therefore, the vertical vector was more intense on the healthy side of the eye. The nystagmus intensity was evaluated using the mean of the vertical nystagmus vectors of both eyes to mitigate the influence of any differences on the evaluation and to compensate for the lack of rotation vectors in the original eye movement data (4).

### Inspection equipment

All Video-nystagmography (VNG) data were obtained from a video nystagmograph instrument: Otometrics ICS Chartr 200 (Otometrics, Denmark).

### Statistical analysis

SPSS 27.0 software was used for statistical processing. Inter-group comparisons of normally distributed data were performed by conducting a t-test. A non-parametric test compared groups when the data did not conform to the normal distribution. Count data were expressed as frequency or percentage, and a chi-squared test was conducted to compare groups. The Kruskal–Wallis test was conducted for pairwise comparisons between multiple groups. Statistical significance was set at a *p* < 0.05.

Apriori analysis: Prescriptions were numbered, and pivot tables were created using WPS. According to the presence or absence of characteristic nystagmus in the transposition test, the values of 0 or 1 were scored (yes = 1, no = 0). The positive frequency of mutation tests was counted, and the mutation tests with a positive frequency of ≥4% were screened. IBM SPSS Modeler 18.0 software analyzed the association rules and network using the Apriori algorithm. The minimum conditional support was set at 10%, the minimum rule confidence was set at 80%, and the maximum number of preceding items was set at five to obtain strong association rules of transposition trials.

## Results

### General information

A total of 2,122 patients with vertigo were admitted to the Department of Encephalopathy, Tongzhou Branch of Beijing Dongzmen Hospital from January 2019 to December 2022. Among these cases, 465 (21.5%) were diagnosed with horizontal canal BPPV (HC-BPPV), 34 (1.6%) with anterior canal BPPV (AC-BPPV), and 1,632 (76.9%) with posterior canal BPPV (PC-BPPV). Of the PC-BPPV cases, 128 (6.0%) were diagnosed with PC-BPPV-cu, and 1,504 (70.9%) were diagnosed with PC-BPPV-ca.

A total of 128 cases of PC-BPPV-cu were included, which comprised 25 men and 103 women, with a men-to-women ratio of 1:4.1. The age range of the patients was 23–88 (57.7 ± 13.8) years. There were 77 patients with right PC-BPPV-cu and 51 with left PC-BPPV-cu. A total of 22 cases were initially observed in the emergency department. The disease course lasted 1 day in 56 cases (43.8%), 2–7 days in 55 cases (43.0%), and >7 days in 17 cases (13.3%).

A total of 128 cases of PC-BPPV-ca were included by conducting a random sampling method, which comprised 31 men and 97 women, with a male-to-female ratio of 1:3.1. The mean age was 55.3 ± 14.9 years (age range, 16–81 years). There were 79 cases of right PC-BPPV-ca and 49 cases of left PC-BPPV-ca. A total of eight cases were first observed in the emergency department. The disease course lasted 1 day in 54 cases (42.2%), 2–7 days in 59 cases (46.1%), and >7 days in 15 cases (11.7%).

Patients in the PC-BPPV-cu group had significantly more emergency visits than those in the PC-BPPV-ca group. Other general data showed no significant differences (*P* > 0.05) (Table 1).

**Table 1:** Comparison of general data between PC-BPPV-cu and PC-BPPV-ca.

**Group**	***n***	**Gender (men/women)**	**Age (years)**	**Course of disease (d)**	**First visit department (emergency/other)**	**Side of the lesion (right/left)**
PC-BPPV-cu	128	25/103	57.7 (48.5, 67.8)	6.9 (1.0, 5.0)	22/106	77/51
PC-BPPV-ca	128	31/97	55.3 (41.0, 67.5)	6.9 (1.0, 5.0)	8/120	79/49
Statistical result		χ^2^ =0.14	F=-1.10	F=0.61	χ^2^ =7.40	χ^2^ =0.07
		*P* = 0.22	*P* = 0.27	*P* = 0.54	*P* = 0.01	*P* = 0.0.80

*P* < 0.05, it has statistical significance.

### Distribution of nystagmus types in PC-BPPV-cu positional testing

A total of nine combinations of nystagmus were observed. The two most frequent patterns were as follows: 387 cases (59.7%) exhibited torsional-upbeat nystagmus and 164 cases (25.3%) exhibited torsional-downbeat nystagmus. The distribution of nystagmus patterns among patients with PC-BPPV-cu varied substantially across different transposition tests (*P* < 0.05). The frequencies of evoked posterior canal excitatory nystagmus (PC-EN) were as follows: affected HH (100%), affected roll test (86.7%), affected DH (75.6%), and SP (36.7%). The frequencies of posterior canal inhibitory nystagmus (PC-IN) were 44.5% in the 45° FDP on the healthy side, 34.4% in the roll test on the healthy side, and 33.6% in the sitting nose-down position (Table 2). Among the 128 patients, 25 exhibited no significant nystagmus in the DH position on the affected side; however, they exhibited PC-EN in the roll test on the affected side. Moreover, nine patients displayed no considerable nystagmus in the roll test on the affected side; however, they exhibited PC-EN in the DH position on the affected side. Among the 128 patients, PC-IN was induced in the 45° FDP without evident nystagmus in 36 patients.

**Table 2:** Distribution of nystagmus types in PC-BPPV-cu position testing.

**Types of nystagmus**	**Affected HH *N* (%)**	**Affected DH *N* (%)**	**Supine position *N* (%)**	**Healthy HH *N* (%)**	**Healthy DH *N* (%)**	**Affected roll test *N* (%)**	**Healthy roll test N(%)**	**45°FDP *N* (%)**	**Sitting nose-down position *N* (%)**	**Summary *N* (%)**
Affected upper pole of the eye to the lower ear +upward	128 (100)	98 (75.6)	47 (36.7)			111 (86.7)	1 (0.8)	2 (1.6)		387 (59.72)
Upward		4 (3.1)	7 (5.5)	1 (0.8)	3 (2.3)	4 (3.1)	2 (1.6)	1 (0.8)		22 (3.40)
Affected upward		1 (0.8)	4 (3.1)			1 (0.8)	1 (0.8)			7 (1.08)
unaffected upper pole of the eye to the lower ear +downward		1 (0.8)			19 (14.8)		44 (34.4)	57 (44.5)	43 (33.6)	164 (25.31)
Downward					11 (8.6)		19 (14.8)	7 (5.5)	12 (9.4)	47 (7.25)
Unaffected upper pole of the eye to the lower ear					1 (0.8)		3(2.3)			4 (0.62)
Affected upper pole of the eye to the lower ear +upward				10 (7.8)					3 (2.3)	13 (2.01)
Unaffected upper pole of the eye to the lower ear +upward					1 (0.8)	3 (2.3)				4 (0.62)
No nystagmus		24 (18.8)	70 (54.7)	117 (91.4)	93 (72.7)	9 (7.0)	57 (44.5)	61 (47.7)	70 (54.7)	
F	24.292									
*P*	0.004									

*P* < 0.05, it has statistical significance.

**Figure 1:**
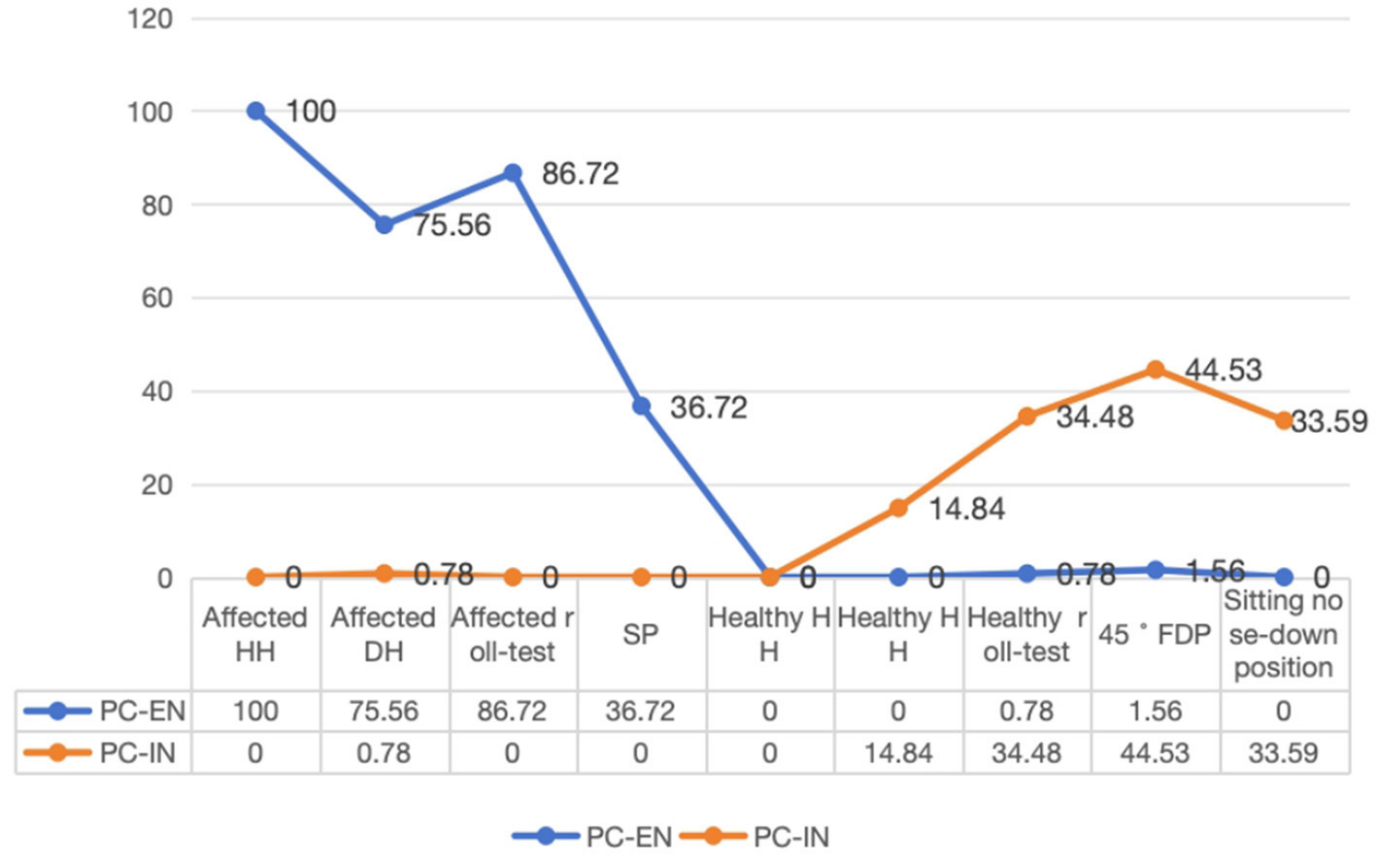
Distribution of positional testing with characteristic nystagmus.

The line chart was created to visually represent the distribution of characteristic nystagmus in PC-BPPV-cu cases based on the results of position testing. The frequencies of PC-EN were as follows: 100% for HH on the affected side, 75.6% for the roll test on the affected side, 75.6% for DH on the affected side, and 36.7% for SP. The PC-IN frequencies were 44.5% for the 45° FDP, 34.5% for the roll test on the healthy side, 33.6% for the sitting nose-down position, and 14.9% for DH on the healthy side (Figure 1).

### Comparison of PC-EN intensity at various positions in PC-BPPV-cu

There were variations in the v-SPV between different positions, which included HH on the affected side, the roll test on the affected side, DH on the affected side, and the SP within the group (Kruskal–Wallis test, H = 47.9, *P* < 0.01). The pairwise comparison revealed significant differences in v-SPV between HH on the affected side, the roll test on the affected side, DH on the affected side, and the SP (all corrected *P* < 0.05). Moreover, a significant difference in v-SPV was observed between the SP and DH on the affected side (adjusted *P* = 0.02). No significant difference in v-SPV was observed between the SP and the roll test on the affected side (adjusted *P* = 0.071). Moreover, there was no significant difference in v-SPV between the DH position and the roll test on the affected side (*P* = 1.0) (*P* > 0.05) (Table 3).

**Table 3:** Comparison of v-SPV in positions where PC-EN was present.

**Position**	**Affected HH**	**Affected DH**	**Affected roll test**	**SP**	**H**	***P***
v-SPV	−6.3 (−11.4, −4.0)	−3.6 (−4.8, −2.6)	−3.6 (−6.1, −2.4)	−5.3 (−8.9, −3.1)	47.9	<0.01

*P* < 0.05, it has statistical significance.

### Comparison of PC-IN intensity at various positions in PC-BPPV-cu

Significant differences were observed in v-SPV on the healthy side in the DH position, the healthy side in the roll-test position, the 45° FDP, and the sitting nose-down position (Kruskal–Wallis, H = 10.0, *P* = 0.02). The pairwise comparison indicated that the nystagmus intensity of the 45° FDP was substantially higher than that of the DH position on the healthy side and the sitting nose-down position (both corrected *p*-values were <0.05). Conversely, no significant difference was observed in v-SPV between the sitting nose-down position and the healthy side in the DH or roll-test positions (*P* > 0.05). Furthermore, there was no significant difference in the intensity of nystagmus between the DH position on the healthy side, the roll test on the healthy side, and the sitting nose-down position (*P* > 0.05) (Table 4).

**Table 4:** Comparison of v-SPV in PC-IN.

**Position**	**Healthy DH**	**Healthy roll test**	**45°FDP**	**Sitting nose-down position**	**H**	***P***
v-SPV	2.4 (1.9, 5.5)	3.6 (2.0, 5.0)	4.2 (3.1, 6.8)	2.6 (1.9, 4.8)	10.0	=0.02

*P* < 0.05, it has statistical significance.

### Intensity comparison of nystagmus between HH on the affected side and the 45° FDP in PC-BPPV-cu patients

In PC-BPPV-cu patients, the average nystagmus intensity was −6.3° (−11.4, −4.0) for HH on the affected side and 4.2° (3.1, 6.8) for the 45° FDP. This difference was significant (Z = −9.1, *P* < 0.01).

### Comparison of the affected-side HH nystagmus intensity in PC-BPPV-cu subgroups with and without nystagmus at the 45° FDP

Within the PC-BPPV-cu group, 57 patients exhibited inhibitory nystagmus induced by the 45° FDP, whereas nystagmus was not induced in 71 cases. The mean v-SPV of nystagmus in the HH position was −10.1° (−11.6, −5.1) in the group with induced nystagmus and −6.6° (−8.8, −3.2) in the group without induced nystagmus, and the difference was statistically significant (*P* = 0.043, *Z* = 2.204).

### Comparison of v-SPV between PC-BPPV-cu and PC-BPPV-ca

A significant difference in nystagmus intensity was observed at the DH position on the affected side between PC-BPPV-ca and multiple positions of PC-BPPV-cu (Kruskal–Wallis test, H = 213.2, *P* < 0.01) (Table 5). Significant differences in nystagmus were observed at the DH position on the affected side between PC-BPPV-ca and PC-BPPV-cu in HH, roll test, DH, and SP (after correction, all *p*-values were <0.05).

**Table 5:** Comparison of nystagmus intensity elicited by PC-BPPV-ca and PC-BPPV-cu at different positions.

	***n***	**Affected HH**	**Affected DH**	**Affected roll test**	**SP**
PC-BPPV-cu	128	−6.3 (−11.4, −4.0)	−3.6 (−4.8, −2.6)	−3.6 (−6.1, −2.4)	−5.3 (−8.9, −3.1)
PC-BPPV-ca	128		−21.9 (−42.6, −9.7)		

### Apriori analysis for correlation analysis of nystagmus at each position of PC-BPPV-cu

The Apriori analysis examined the association between each position when PC-EN or PC-IN occurred. Given that characteristic nystagmus on the affected side of HH is the diagnostic criterion for PC-BPPV-cu, the focus was primarily on positions strongly associated with HH on the affected side.

**Figure 2:**
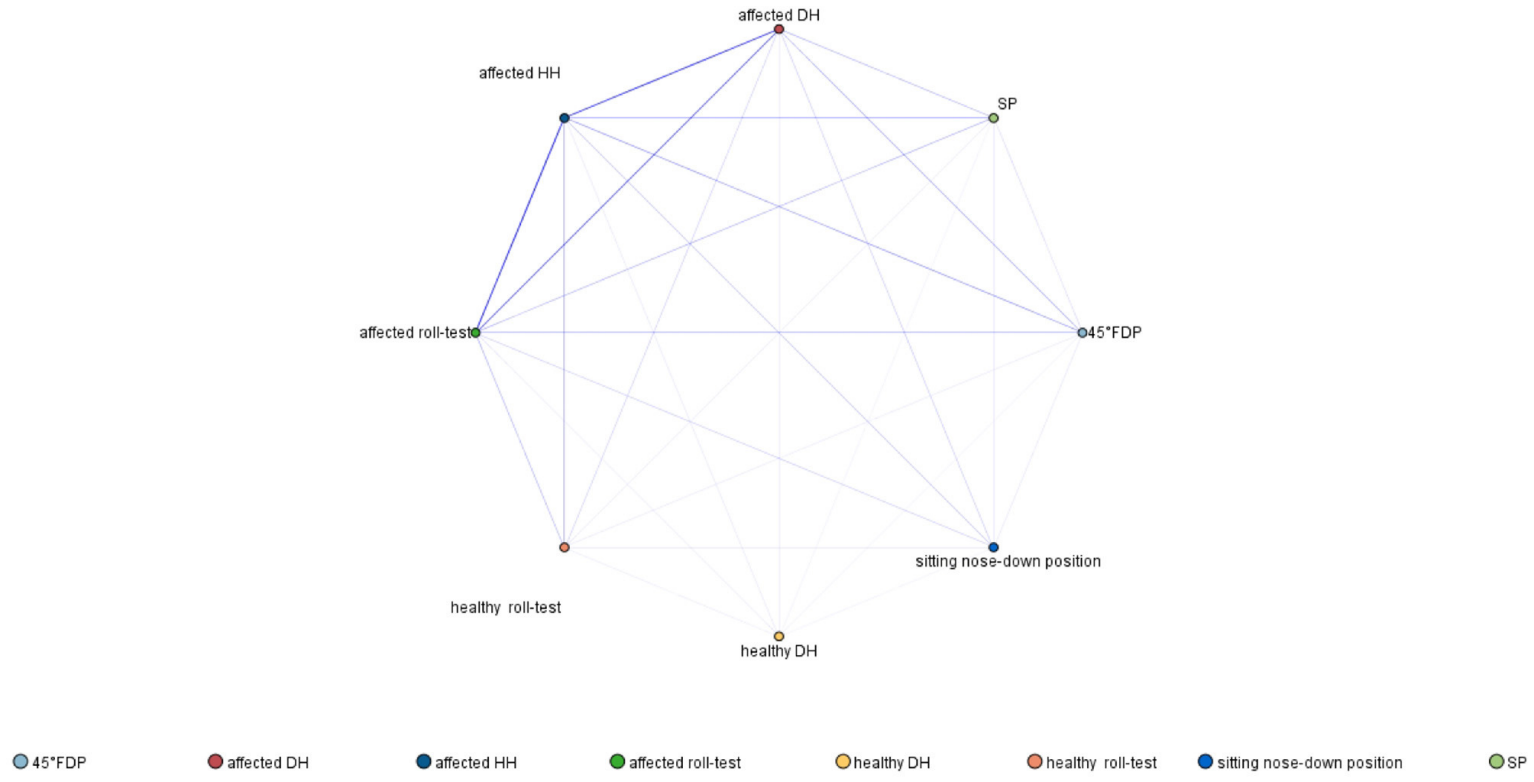
Association rule network diagram of PC-BPPV-cu demonstrating positions associated with excitation and inhibition of the posterior canal (with a frequency of ≥4%).

From the findings, seven rules were identified, with two showing strong associations, with the highest support observed in the affected roll test → affected side (84.4%). There were 18 strong association rules, with the highest support observed in the affected roll test and affected DH → affected HH (65.6%). In addition, sixteen rules were identified, with four strong association rules. The rule with the highest support was the 45° FDP, the affected roll test, and affected DH → affected HH (32.0%). Moreover, seven rules were observed, along with five strong association rules. The combination SP, the affected roll test, the 45° FDP, and affected DH → affected HH had the highest support (17.2%). The network diagram of association rules is shown in Figure 2. Thicker and darker lines represent more robust associations. Notable connections include affected HH with the affected roll test, affected HH with affected DH, affected HH with the 45° FDP, and affected HH with SP.

## Discussion

### General characteristics of PC-BPPV-cu

This study examined 2,122 cases of BPPV. The distribution of various BPPV types aligned with the epidemiological patterns of the disease (5). PC-BPPV-cu is considered a rare subtype of PC-BPPV (2). The Chinese guidelines for diagnosing and treating BPPV reported that the proportion of this disease in BPPV cases was approximately 6.3% (5), while other studies reported the proportion to be7.2% (6). In this study, the incidence of PC-BPPV-cu was 6.03%, consistent with previous studies.

In this study, the male-to-female ratio among patients with PC-BPPV-cu was 1:4.1, predominantly affecting middle-aged and older individuals, aligning with prior studies (7). BPPV is more prevalent in women, attributed to various factors such as estrogen levels, bone mineral density, and susceptibility to autoimmune diseases (8, 9). Compared with PC-BPPV-ca, more patients with PC-BPPV-cu opt for emergency department visits, likely due to the prolonged duration of vertigo and the more comprehensive range of vertigo-inducing positions associated with this condition. Few patients with PC-BPPV-cu experience persistent vertigo and clinical manifestations that resemble acute vestibular syndrome, which leads to initial misdiagnosis of cupulolithiasis.

### Characteristics of nystagmus in PC-BPPV-cu

A limited number of studies have investigated the characteristics of nystagmus in PC-BPPV-cu. Ichijo (10) analyzed the clinical and nystagmus characteristics in 30 patients with PC-BPPV-cu. The findings revealed torsional and vertical (up beating) nystagmus lasting over 1 min in the supine and affected ear-down positions; conversely, torsional and vertical (down beating) nystagmus was observed in the nose-down position, lasting for a similar duration. The present study employed a more comprehensive array of positional tests.

In our study, the most common positional nystagmus pattern of PC-BPPV-cu was torsional-up beating (59.7%), followed by torsional-down beating (25.3%). Torsional-up beating indicates PC-EN, while torsional-down beating indicates PC-IN.

At the affected HH position, the long axis of the posterior semicircular cupula becomes perpendicular to gravity, experiencing maximal gravitational impact, which results in the strongest nystagmus (1). Our study corroborated these findings by observing the highest nystagmus intensity at the HH position, aligning with its anatomical traits.

In our investigation, the v-SPV of positional nystagmus at the affected HH position surpassed that observed in other positions with PC-EN (all *P* < 0.05), thereby supporting anatomical alignment. However, Wang et al. (7) analyzed the clinical data of 46 patients with PC-BPPV-cu, which revealed slower SPV of induced nystagmus at the DH position compared to the HH position. The discrepancy in nystagmus intensity at the HH position may relate to the angle of head elevation from the bed surface.

In our methodology, the HH maneuver did not involve lifting the patient's head from the bed, contrasting with the approach described in the Fifth edition of The Neurology of Eye Movements (4). Wang et al. (7) employed a different technique where the head was elevated approximately 30° in flexion from the SP. Based on this technique, the HH position without head elevation was theorized to elicit the strongest positional nystagmus in PC-BPPV-cu. Future studies could explore the nystagmus characteristics between supine HH and HH with head elevation.

PC-EN in PC-BPPV-cu was observed in the affected side roll test, DH position, and SP, with occurrence rates of 86.7%, 75.6%, and 36.7%, respectively. The roll test on the affected side exhibited the highest probability of inducing PC-EN, attributed to the angle between the long axis of the cupula of the affected side and gravity in this position. Notably, in 25 cases of PC-BPPV-cu, nystagmus was not induced in the affected DH position, but excitatory nystagmus was evoked in the roll test on the affected side. Since the HH position is not routinely included in positional testing, the affected roll-test position may be the initial site where PC-EN is sometimes observed.

**Figure 3:**
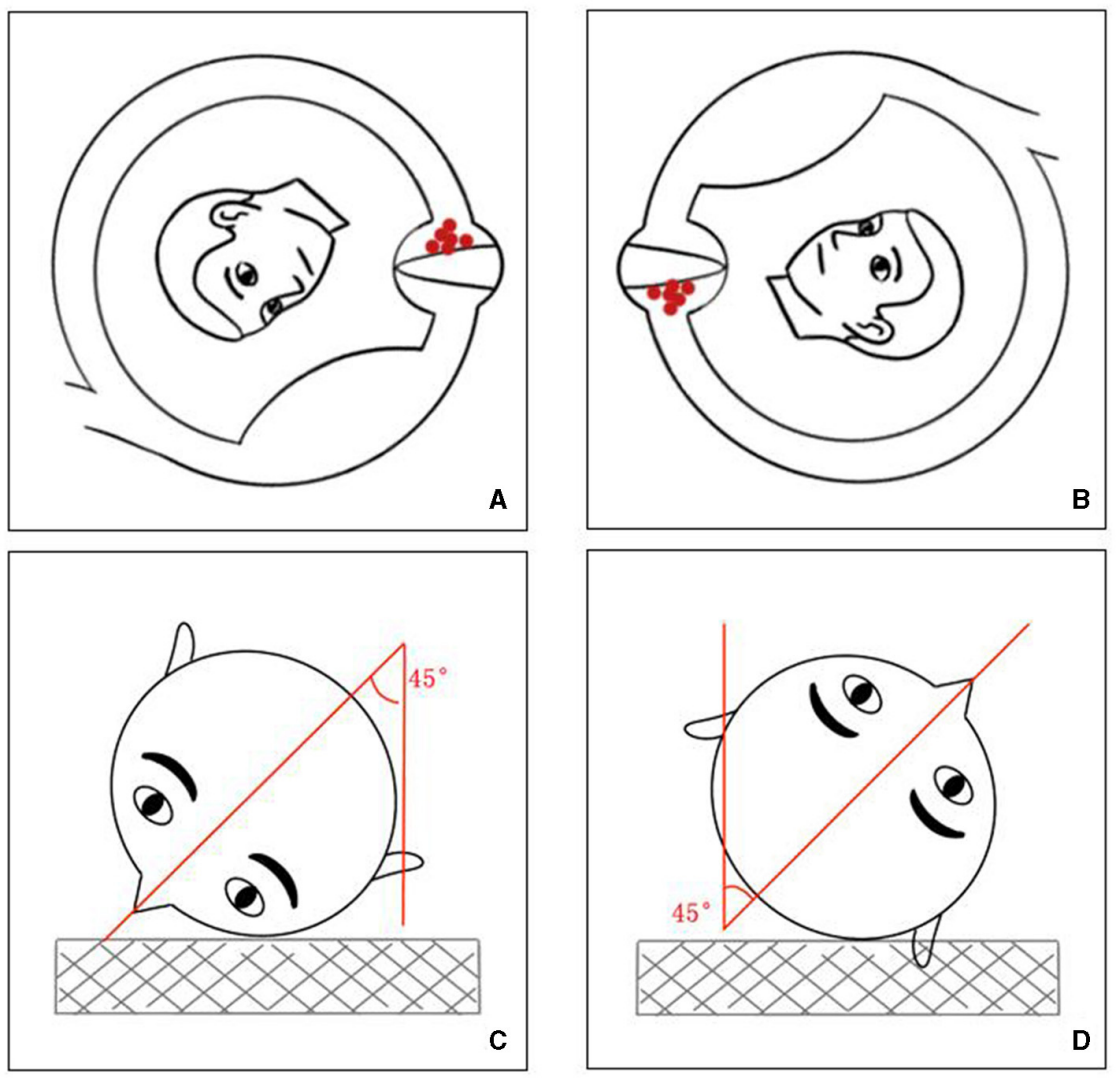
Schematic representation of the 45° FDP. **(A, C)** Depiction of the head's orientation relative to the examination bed and the position of the semicircular canal's cupula at the 45° FDP. **(B, D)** Illustration of the head's orientation relative to the examination bed and the position of the semicircular canal's cupula at HH.

In cupulolithiasis, a characteristic inverted nystagmus emerges when the head is turned 180°, showcasing its uniqueness (5). In our investigation, the position that elicited the strongest nystagmus due to PC-BPPV-cu within the plane of the affected PC was the HH position, which, when flipped 180°, corresponded to the 45° FDP on the healthy side (Figure 3). Inhibitory nystagmus was observed in the 45° FDP, the roll test on the healthy side, and the nose-down position, with the 45° FDP showing the most significant nystagmus intensity.

Not all of patients with PC-BPPV-cu had exhibited characteristic nystagmus at the 45° FDP. This observation could be attributed to the weaker inhibitory nystagmus. While excitatory nystagmus was consistently observed at the HH position in all the cases, the inhibitory nystagmus was weak, and it may not manifest in cases with weaker nystagmus. This hypothesis was verified by comparing the vertical angular velocity of excitatory nystagmus in the HH position between patients positive for nystagmus and those negative for nystagmus at the 45° FDP, corroborating our conjecture. Specifically, the vertical angular velocity at HH was lower in patients negative for nystagmus at the 45° FDP.

Moreover, 36 patients with PC-BPPV-cu failed to elicit significant nystagmus in the nose-down position; however, they exhibited inhibitory nystagmus at the 45° FDP. This phenomenon may stem from limited neck movement in most patients, and the head's bowing angle in the sitting position is frequently <90°. Consequently, the 45° FDP proves superior to the nose-down position in detecting reverse nystagmus associated with PC-BPPV-cu.

Furthermore, only 44.5% of PC-BPPV-cu cases triggered PC-IN at the 45° FDP, which exceeded the occurrence at the nose-down position. The lower incidence of inhibitory nystagmus, as compared to its excitatory counterpart, could be attributed to its weaker nature, rendering it difficult to induce in cases with milder nystagmus.

When nystagmus intensity at the HH position was compared between cases with nystagmus and those without at the 45° FDP, more vigorous nystagmus intensity was observed in the latter group, which reinforced our earlier speculation. The disparity in nystagmus intensity between the HH position and the 45° FDP aligns with Ewald's third law, highlighting the contrasting excitatory and inhibitory potentials. Ichijo (11) reported a similar trend in PC-BPPV-cu cases, where nystagmus intensity was more significant in the head-hanging position than in the nose-down position, which further highlighted the distinction between excitatory and inhibitory potentials. However, the study lacked nystagmus data from the HH position.

PC-BPPV-cu showed the strongest nystagmus at the HH position on the affected side, while nystagmus intensity at the DH position was significantly weaker, which was consistent with the findings of other studies (3). According to Pascal's principle, the pulling force on the cupula caused by the movement of free otolith particles in the membranous labyrinth is significantly more potent than that caused by gravity, being up to 15 times more significant (12). Consequently, the positional nystagmus PC-BPPV-cu is weaker and more persistent than PC-BPPV-ca.

### Clinical application of positional testing for PC-BPPV-cu diagnosis

The apriori correlation analysis showed the highest correlation between the affected side roll test and HH position nystagmus, followed by DH position nystagmus. The strongest correlation between inhibitory nystagmus and HH nystagmus was at the 45° FDP on the healthy side.

According to the association rule network, aside from the HH position on the affected side, the roll test on the affected side is the most likely position to induce excitatory nystagmus in PC-BPPV-cu. On the other hand, the 45° FDP on the healthy side is the most likely position to induce reverse nystagmus. The association rule network diagram indicates strong associations between the affected roll test, 45° FDP, and HH positions. Therefore, in patients with PC-BPPV-cu, the following positional tests (HH, affected roll test, and 45° FDP) are more likely to induce characteristic nystagmus.

Since HH is not a routine position test item in clinical practice, the first position where rotational-up beating nystagmus is observed in some PC-BPPV-cu cases may be the affected-side roll-test position. Based on this observation, the best supplementary positional test scheme for identifying PC-BPPV-cu involves completing the HH and flipping to the 180° (45° FDP) position on the affected side while observing for inhibitory or excitatory nystagmus of the responsible semicircular canal.

Previous studies have reported cases of canal jam in the PC (13). Conditions, such as canal jam, should be considered for PC-BPPV-cu cases without PC-IN.

## Conclusion

The positional test is a pivotal component in the clinical examination of vertigo. In PC-BPPV-cu, the strongest excitatory nystagmus of the responsible semicircular canal was elicited at the HH position on the affected side. In contrast, the most potent inhibitory nystagmus was evoked at the 45° FDP on the healthy side. In some instances of PC-BPPV-cu, nystagmus was not triggered in the DH position; however, the vertical rotational nystagmus could be induced in the roll test position. Clinicians should consider incorporating the HH position and the 45° FDP to enhance the diagnostic accuracy of PC-BPPV-cu.

## Limitations and prospects

Angular velocity data for the rotational component of positional nystagmus were lacking in most BPPV cases at our center due to the inherent constraints of retrospective studies and examination equipment. Consequently, only the vertical component served as an index for evaluating the intensity of nystagmus, overlooking the contribution of the rotational element.

Prospective studies are currently being conducted to further explore the variations in nystagmus intensity and null plane characteristics of PC-BPPV-cu across different locations.

## Data availability statement

The raw data supporting the conclusions of this article will be made available by the authors, without undue reservation.

## Ethics statement

The studies involving humans were approved by Ethics Committee of Dongzhimen Hospital Affiliated to Beijing University of Chinese Medicine. The studies were conducted in accordance with the local legislation and institutional requirements. The Ethics Committee/institutional review board waived the requirement of written informed consent for participation from the participants or the participants' legal guardians/next of kin due to the retrospective nature of the study.

## Author contributions

JW: Writing – original draft, Writing – review & editing, Conceptualization, Data curation, Formal analysis, Investigation, Methodology, Software. YZ: Conceptualization, Writing – review & editing, Supervision, Writing – original draft. WX: Data curation, Investigation, Writing – review & editing. HM: Investigation, Supervision, Writing – review & editing. LH: Data curation, Writing – review & editing. BZ: Conceptualization, Writing – original draft. LS: Methodology, Data curation, Writing – review & editing.
